# Supplementary material for: ESR1 Methylation Measured in Cell-Free DNA to Evaluate Endocrine Resistance in Metastatic Breast Cancer Patients
Source: Int J Mol Sci. 2022 May 18;23(10):5631. doi: 10.3390/ijms23105631 (PMC9142900; doi:10.3390/ijms23105631)
Supplement: Supplementary file 1 [file ijms-23-05631-s001.zip › ijms-1688245-supplementary.pdf]

Supplemental data

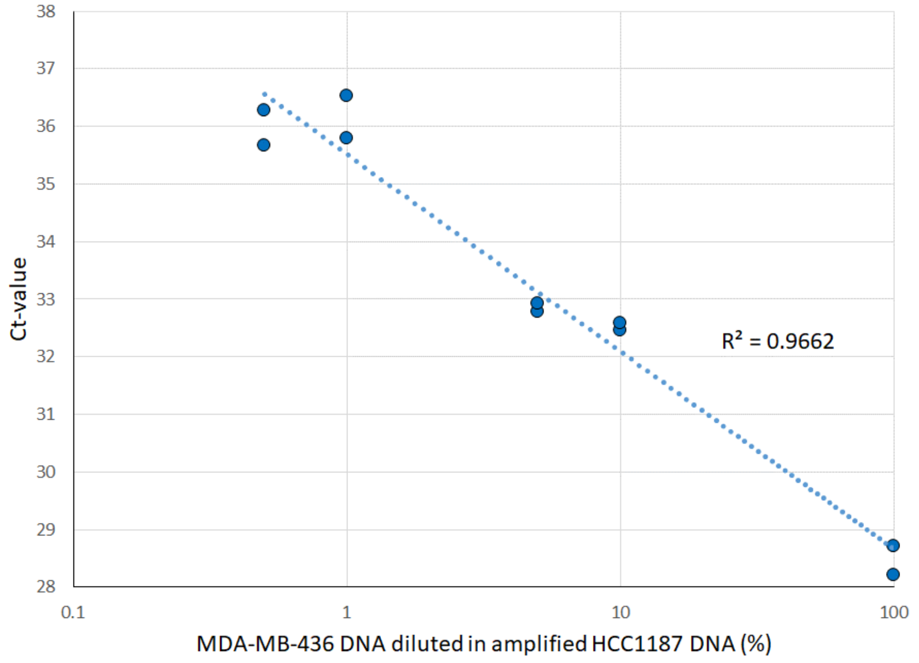

Supplemental Figure S1. Dilution series methylated DNA in unmethylated DNA

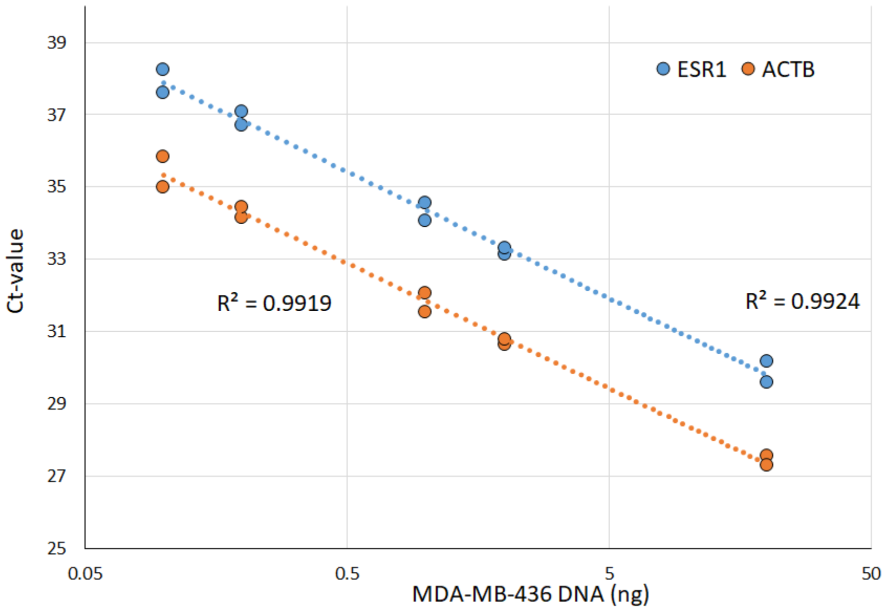

Supplemental Figure S2. Duplex-assay in input-limiting dilution series

Supplemental Tables

Table S1. Age of samples from patients and healthy donors that were included in our study and of which *ESR1* methylation was determined successfully.

	Cohort 1			Cohort 2		
	**Patients n=31**	**Healthy donors n=18**		**Patients n=17**	**Healthy donors n=8**	
**Median age (range) in years**	66 (47-88)	58 (55-74)	p=0.009	66 (50-84)	61 (55-70)	p=0.391

Table S2. Location and Cg probes of *ESR1* promoters.

Cg probe	Promoter	Chromosome	Start (hg38)	End (hg38)	Gene Symbol
cg08415493	C	chr6	151803680	151803681	ESR1
cg20893956	C	chr6	151805601	151805602	ESR1
cg24764793	C	chr6	151805610	151805611	ESR1
cg07746998	C	chr6	151805650	151805651	ESR1
cg21157690	C	chr6	151805760	151805761	ESR1
cg17264271	C	chr6	151805803	151805804	ESR1
cg20627916	B	chr6	151807193	151807194	ESR1
cg07671949	B	chr6	151807203	151807204	ESR1
cg23164938	B	chr6	151807231	151807232	ESR1
cg23165623	B	chr6	151807276	151807277	ESR1
cg21614759	B	chr6	151807291	151807292	ESR1
cg19411146	B	chr6	151807336	151807337	ESR1
cg21950534	B	chr6	151807348	151807349	ESR1
cg11813455	B	chr6	151807380	151807381	ESR1
cg24900983	B	chr6	151807393	151807394	ESR1
cg05171584	B	chr6	151807400	151807401	ESR1
cg23467008	B	chr6	151807402	151807403	ESR1
cg22839866	B	chr6	151807449	151807450	ESR1
cg23009221	B	chr6	151807453	151807454	ESR1
cg13612689	B	chr6	151807499	151807500	ESR1
cg27316393	B	chr6	151807540	151807541	ESR1
cg00655307	A	chr6	151807608	151807609	ESR1
cg01777019	A	chr6	151807670	151807671	ESR1
cg15980539	A	chr6	151807901	151807902	ESR1
cg11251858	A	chr6	151807901	151807902	ESR1

Table S3. Primer sequences of our ESR1 methylation assay. FAM: fluorescein amidite, EQ: eclipse quencher, MGB: minor groove binding moiety, QXL670: dark quencher.

**Name**		**Sequence 5′ → 3′**
ESR1 Promoter A	Forward	GTTTTGGGATTGTATTTGTTTTCG
	Reverse	GCCGACACGCGAACTCTAA
	Probe	**FAM**-CGATAAAACCGAACGACC-**EQ**-**MGB**
ACTB	Forward	AACCAATAAAACCTACTCCTCCCTTAA
	Reverse	TGGTGATGGAGGAGGTTTAGTAAG
	Probe	**Cy5**-ACCACCACCCAACACACAATAACAAACACA-**QXL670**
